# The Relationship between Workplace Ostracism, TMX, Task Interdependence, and Task Performance: A Moderated Mediation Model

**DOI:** 10.3390/ijerph17124432

**Published:** 2020-06-20

**Authors:** Yang Woon Chung

**Affiliations:** Department of Business Administration, University of Suwon, 17 Wauan-gil, Bongdam-eup, Hwaseong-si, Gyeonggi-do 445-743, Korea; jywchung@suwon.ac.kr; Tel.: +82-31-220-2299

**Keywords:** workplace ostracism, team–member exchange, task interdependence, task performance, moderated mediation

## Abstract

*Background*: Social interactions among employees are essential for individual performance as they provide various job-related information and feedback as well as social and emotional support. Tasks have become interdependent among organizational members, allowing teamwork to generally become an organizational norm. Consequently, it is pertinent that employees maintain favorable working relationships with other organizational members because workplace ostracism has become an organizational concern. Although recent studies have examined numerous psychological mechanisms that associate ostracism with workplace outcomes, studies have been limited in exploring practical facets that link the relationship. Thus, this study examined the mediating effect of team–member exchange for workplace ostracism and task performance and the moderating effect of task interdependence in influencing the relationship. *Methods*: Data were collected using a two-wave design and sampled 242 full-time employees in South Korea. The hypotheses were tested with hierarchical regression analyses. *Results*: Team–member exchange was found to mediate the relationship between workplace ostracism and task performance and task interdependence moderated the mediated relationship. *Conclusions*: The results suggest that being ostracized negatively influences the quality of the relationship between team-members which then affects individual performance. In addition, the conditional indirect effect for ostracism on task performance was significant when task interdependence was high, while not significant when it was low, thus moderating the mediated relationship.

## 1. Introduction

Organizations require social interactions among organizational members to perform effectively. As work has become more interdependent and the frequency of teamwork has increased, social interactions among organizational members have become critical aspects on individual and organizational performance [1,2]. Establishing and maintaining positive interpersonal relationships are important factors in performance, but unfortunately, the workplace has become a social context where ostracism occurs [3]. Ostracism is when an individual or group does not engage in actions that include organizational members when it is socially appropriate to do so [4]. Subsequently, ostracism has been found to have numerous detrimental effects on individuals and organizations as studies have found workplace ostracism to reduce organizational commitment, job satisfaction, and organizational citizenship behavior [5,6], and increase negative workplace behaviors such as counterproductive behavior, harassment, and aggressive behavior [7,8].

Robinson, et al.’s [4] conceptualized framework suggests several mediating and moderating variables that can elaborate the effects of workplace ostracism on workplace outcomes. They argued that psychological and pragmatic effects of ostracism can link ostracism and behavioral outcomes and that the underlying mechanisms have been overlooked in the literature. They suggested that pragmatic effects that influence task-related resources, relationships, and information can associate the relationships between workplace ostracism and job performance, organizational citizenship behavior, and deviant behavior. Furthermore, they explained that task interdependence, identification, and status can moderate the relationships between workplace ostracism and behavioral outcomes. Although recent studies have investigated the mediating mechanisms for the relationships between workplace ostracism and workplace behaviors, most of the studies have focused on the psychological facets such as job engagement, self-esteem, and person–organization fit [7,9,10,11], while studies have yet to explore the more practical aspects such as interpersonal conflict [12].

Therefore, this study aims at bridging the gaps within the extant literature as it jointly investigates the mediating effect of team–member exchange and the moderating effect of task interdependence on the relationship between workplace ostracism and task performance. Furthermore, the study goes beyond the moderating effect and proposes that task interdependence will moderate the mediated relationships between workplace ostracism, team–member exchange, and task performance. In other words, the indirect effects of workplace ostracism on task performance via team–member exchange will be enhanced when task interdependence is high in comparison with when task interdependence is low.

## 2. Hypothesis Development

Team–member exchange (TMX) refers to how an individual perceives one’s exchange relationship with other members within a group. TMX explains an individual’s willingness to help other members, share ideas and feedback, and how readily information, help, and recognition are received from other group members, thereby forming a reciprocal relationship [13]. The quality of the TMX relationship refers to the effectiveness of an individual’s working relationship with other group members. In order to maintain positive workplace relationships and engage in positive workplace behaviors, individuals need to regulate themselves to maintain the persistence and effort that are necessary to perform tasks and maintain the good image of organizational citizens [14,15]. Self-awareness and the ability to understand long-term consequences related to one’s behaviors are pertinent facets of self-regulation. In this regard, workplace ostracism will be negatively related to TMX because ostracism has been argued to result in maladaptive responses since ostracism negatively influences one’s cognitive state. Ostracism impairs logical reasoning and affects the ability to self-regulate, reduces self-awareness, emphasizes the present state, and has a lack of concern for long-term goals [16,17]. Further, ostracism will negatively impact the ability to adapt one’s behavior to comply with social norms and to achieve goals, thus being likely to engage in maladaptive behaviors [17]. Social exchange theory and the norm of reciprocity can further strengthen the relationship between workplace ostracism and TMX, as ostracized individuals will be less likely to be motivated to engage in prosocial behaviors toward organizational members due to their aversive perceptions regarding their interpersonal relationships.

When individuals identify themselves to be part of a group, the exchange relationships provide individuals with greater opportunities to meet performance expectations [13]. TMX explains the relationships with others within a group and that the quality of the relationship can significantly affect an individual’s task performance. As interpersonal relationships are good sources of resources, individuals that experience low TMX will be likely to have limited exchanges which then can negatively affect the completion of their tasks. In contrast, when TMX is high, relationships will involve more frequent exchanges that can increase one’s resources and support which then can extend beyond what is needed for completing one’s task [18]. TMX can increase task performance because high-quality TMX relationships promote the willingness to assist other members and share information, ideas, and feedback with them [13,18]. Tse and Dasborough [19] found that high-quality TMX relationships resulted in numerous task-oriented exchanges such as work-related problem solving and frequent work communication which can subsequently enhance one’s performance.

According to the conservation of resources theory [20], resources are valuable to people and they attempt to protect and sustain them. Resources are objects, personal characteristics, conditions, or energies that are valued or serve as a means for the attainment of these objects, personal characteristics, conditions, or energies [20]. Research shows that coworker support greatly influences an individual’s effectiveness in the workplace. Support from coworkers can be considered to be a job resource and since resources are motivational in nature, resources can greatly contribute to work engagement. In contrast, when there is a loss of resources, a perceived threat of loss, or a lack of resource gain following an investment of resources, performance can be negatively affected [20]. Furthermore, when employees lack resources, they will attempt to protect their remaining resources by depersonalization and decreasing their work engagement and performance efforts, therefore explaining that workplace ostracism can reduce an individual’s resources which then negatively affects performance.

It is important that employees know how to regulate their behaviors when experiencing negative workplace events such as workplace ostracism in order to maintain persistence and effort to successfully perform and complete their tasks. However, as workplace ostracism has been suggested to cause maladaptive responses, an ostracized individual’s ability to self-regulate or adapt behavior to comply with social norms will be negatively affected, therefore being likely to result in maladaptive behaviors [17]. Hitlan and Noel [21] argued that when an individual is excluded, rejected, ignored, or ostracized by another individual or group at work, these behaviors hinder an individual’s ability to complete one’s tasks that are required for successful job performance. In this regard, ostracized individuals will be less likely to engage in behaviors that promote positive and social interpersonal relationships which then can negatively affect an individual’s completion of tasks, thus proposing the following:

**Hypothesis** **1.** *TMX will mediate the relationship between workplace ostracism and task performance.*

Task interdependence is the extent to which an individual needs resources such as information, materials, and support from other members to be able to do his or her job. High task interdependence requires individuals to have increased communication, cooperation, and coordinated behaviors in order to accomplish their goals. In contrast, when task interdependence is low, individuals can perform and complete their tasks individually without much interaction with other organizational members. Although task interdependence is usually explained by the group level of analysis, task interdependence can also be defined at the individual level as it refers to the perceptions about the structural relationships among group members [22]. In this regard, task interdependence is referred to be experienced or perceived by an individual, thereby influencing individual outcomes due to the effects of social facilitation or the motivation of individuals to maintain a positive image in the presence of potential evaluators [23]. Previous studies found task interdependence to be positively related to individual outcomes such as felt responsibility of work [24] and the frequency of how often individuals sought help for performance-related problems [25].

As work is becoming more interdependent and organizations are frequently implementing teamwork, the relationship between TMX and task performance can vary on the degree of task interdependence. For instance, when task interdependence is high, there will be a greater need for individuals to communicate, support, and help each other. The interactions between the individuals will be likely to increase and result in enhancing the relationship between TMX and task performance, therefore, task interdependence can moderate the relationship. Similarly, Alge, et al. [26] found TMX to be positively associated with decision-making effectiveness when task interdependence was high. However, the current study goes beyond the moderating effect and proposes that task interdependence will moderate the mediated relationship between workplace ostracism, TMX, and task performance. Task interdependence will be able to constrain the mediated relationship because ostracized individuals will be likely to avoid direct interpersonal interactions and decrease their social behaviors toward other organizational members. In this regard, the indirect effects of workplace ostracism on task performance via TMX can be alleviated when task interdependence is low as it can decrease the discomfort of social interactions when trying to complete one’s task. In contrast, when task interdependence is high, the indirect effects of workplace ostracism on task performance via TMX will further result in detrimental outcomes due to the need for more frequent interactions that ostracized individuals do not prefer, therefore proposing the following:

**Hypothesis** **2.** *Task interdependence will moderate the strength of the mediated relationships between workplace ostracism and task performance via TMX such that the mediated relationship will be stronger under high levels of task interdependence than under low levels of task interdependence.*

## 3. Materials and Methods

### 3.1. Sample

The study was conducted in South Korea and the self-reported surveys were administered at two data collection points. The questionnaires were sent out to full-time employees in 5 organizations within the telecom, IT, and financial industries. The first questionnaires (T1) consisted of demographic information and workplace ostracism and were given to 434 employees and 366 questionnaires were returned (84% response rate). Out of the 366 questionnaires, 343 were usable and cases with missing data were excluded. A two-month interval was used between the first and second questionnaires in order to reduce the biases relating to single sources and common methods as recommended by Podsakoff, et al. [27]. The second questionnaires (T2) measured TMX, task interdependence, and task performance and were sent to 343 employees and 281 were returned (82% response rate). Out of the 281 questionnaires, 242 were usable due to missing data.

### 3.2. Measures

The questionnaires were translated into Korean and were later back-translated into English by two fluent bilingual persons in order to validate the quality of the translations [28]. The questionnaire used a 7-point Likert scale from 1, “strongly disagree,” to 7, “strongly agree”, for all of the measures.

Ferris et al.’s [5] ten-item scale was used to measure workplace ostracism. To measure reliability, McDonald’s ω was estimated for the study and the reliability of this scale was 0.97. Sample items are: “Others at work shut you out of the conversation,” and “Others left the area when you entered.”

TMX was measured with Seers, Petty, and Cashman’s [29] ten-item scale and the reliability of this scale was 0.85. Sample items are: “I often make suggestions about better work methods to other team members,” and “Other members of my team usually let me know when I do something that makes their jobs easier.”

Task interdependence was measured with Pearce and Gregersen’s [24] six-item scale and the reliability of this scale was 0.78. Sample items are: “My own performance is dependent on receiving accurate information from others,” and “The way I perform my job has a significant impact on others.”

Task performance was measured with Williams and Anderson’s [30] seven-item measure and the reliability of this scale was 0.95. Sample items are: “I perform tasks that are expected from me,” and “I engage in activities that will directly affect my performance evaluation.”

Gender, age, education, position, tenure, and team tenure were controlled for as they could be potential differentiates.

## 4. Results

Table 1 shows the descriptive statistics, correlations, and reliability estimates for the study variables. To test the hypotheses, hierarchical multiple regressions were conducted and the control variables were included for the analyses.

Hypothesis 1 proposed that TMX will mediate the relationship between workplace ostracism and task performance. The mediation analysis was conducted with Preacher and Hayes’s [31] technique which includes the multistep approach by Baron and Kenny [32] but also includes bootstrapping which does not assume that the indirect effects are normally distributed. The hypotheses were tested using the macro PROCESS and the results in Table 2 show that workplace ostracism was related to TMX (β = −0.33, *p* < 0.001) and TMX was related to task performance when controlling for workplace ostracism (β = 0.28, *p* < 0.001). Further, the indirect effect path was significant (−0.09, *p* < 0.001). Furthermore, the bootstrap results with a bootstrapped 95% CI around the indirect effect did not contain zero for task performance (−0.17, −0.05), thus supporting Hypothesis 1.

**Figure 1:**
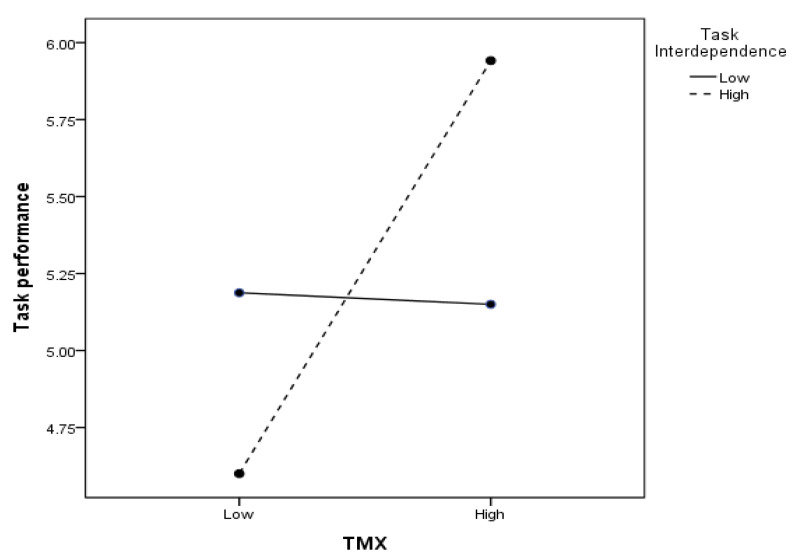
The moderating effects of task interdependence on the relationship between team–member exchange (TMX) and task performance.

Hypothesis 2 proposed that task interdependence will moderate the mediated relationship between workplace ostracism and task performance via TMX. First, the moderated effects of task interdependence for the relationship between TMX and task performance were estimated. As shown in Table 3, task interdependence significantly moderated the relationship (β = 0.11, *p* < 0.05, f^2^ = 0.03). The interaction effects were then plotted at one standard deviation above and below the mean. Figure 1 saliently depicts that task interdependence influences the relationships between TMX and task performance, as high levels of task interdependence greatly increased task performance, while low levels of task interdependence slightly decreased task performance.

Afterwards, to test for moderated mediation, Preacher, Rucker, and Hayes’s [33] regression-based method was followed in order to estimate the conditional indirect effects of the moderator. High and low levels of the moderator were operationalized at one standard deviation above and below the mean [33]. Table 4 depicts that the conditional indirect effect for workplace ostracism on task performance was significant when task interdependence was high (workplace ostracism = −0.13, *p* < 0.00), while not significant when task interdependence was low (workplace ostracism = −0.05; *ns*), thus suggesting that the indirect effects of workplace ostracism on task performance through TMX significantly differ by levels of task interdependence.

## 5. Discussion

The social context within organizations can significantly influence an employee’s workplace attitudes and behaviors. Organizational members can affect how one feels about his or her work and other work-related facets and the study found that being ostracized by organizational members negatively affects the quality of exchange between team members. Being ostracized can negatively influence an individual’s cognitive state which then affects the ability to self-regulate one’s workplace perceptions and behaviors. Subsequently, ostracized individuals will be less likely to engage in positive social behaviors such as providing suggestions that can assist others with their work methods and completion of their work. Ostracism negatively affects the quality of a social exchange, thus suggesting that ostracized employees form negative reciprocal relationships with other team members. In this regard, other team members will also be less likely to have favorable perceptions about ostracized individuals and be less likely to engage in prosocial behaviors toward them, therefore further exacerbating the quality of exchanges between organizational members. In addition, ostracized individuals will tend to avoid direct communication and interpersonal interactions with their team members. Hence, due to the negative perceptions regarding interpersonal exchanges, ostracized individuals will tend to engage in withholding behaviors such as failing to provide coworkers with important information.

Although research has been sparse on examining the relationship between TMX and individual performance, the study findings were consistent with prior research [13,18] as TMX was found to be positively related to task performance. High levels of TMX explain that individuals with close working relationships with other team members enhance one’s task performance because positive TMX relationships rely more on frequent communication of sharing information, ideas, and feedback with each other. Positive TMX relationships further allow team members to help each other by also engaging in extra-role behaviors that can assist others to accomplish their tasks. Therefore, when an individual has more work-related resources, it will be highly likely that one’s performance will increase due to the positive and helpful exchanges with other organizational members.

Workplace ostracism may negatively affect an individual’s task performance due to the loss of resources. According to the conservation of resources theory, individuals that lack resources will attempt to protect their remaining resources by depersonalization, reductions in their work engagement, and a decrease in their performance efforts. When an individual lacks valuable resources such as information, feedback, and social support, the quality of one’s performance will be likely to decrease. Since workplace ostracism adversely affects interpersonal behavior, it can also negatively affect individual performance [5] because of the lack of human resource exchanges. In addition, ostracism negatively influences an individual’s self-regulatory ability [17] and is argued to lower cognitive performance [16]. Similarly, Williams [34] suggested that ostracism influences the basic needs for belonging, self-esteem, control, and meaningfulness and that ostracized individuals will be more likely to engage in behaviors that help recoup their threatened needs. Thereby, ostracism can negatively affect the quality of work and ability to complete work tasks.

As work has become team-based, individuals are required to closely collaborate with other organizational members to effectively complete one’s tasks. Sharing information and resources and providing support are essential aspects that can significantly improve one’s job tasks. In this regard, the study results clearly suggest that an individual’s task performance is not only affected by the quality of the relationships between team members, but it can further be influenced by the level of effort and skills of other organizational members. Teamwork requires organizational members to work closely with one another, thus task interdependence is essential in successfully accomplishing one’s tasks. Since work is now requiring more frequent communication, cooperation, and coordination among organizational members, task interdependence emphasizes the need for organizational members to coordinate in a more effective manner and to be able to assist other organizational members to complete their tasks. Thus, the study found task interdependence to moderate the mediating relationships between workplace ostracism, TMX, and task performance. To further elaborate, the study found TMX to mediate the relationship between workplace ostracism and task performance when ostracized individuals perceived high task interdependence but not when they perceived low task interdependence. This finding suggests that when ostracized individuals have to interact more frequently with other organizational members for their work tasks, it further exacerbates the negative effects of workplace ostracism on task performance via TMX. Therefore, the study demonstrated that the indirect effect of workplace ostracism on task performance through TMX significantly differs by the levels of task interdependence.

### 5.1. Implications

Past research has primarily focused on the psychological aspects for associating workplace ostracism and behavioral outcomes, while the pragmatic effects have been overlooked [4]. The current study investigated how the quality of relationships between team members would be influenced by workplace ostracism and found TMX to associate the relationship between workplace ostracism and task performance. The study results provide empirical evidence that task-related resources, quality of relationships, and information are influenced by workplace ostracism which then influences performance. This finding emphasizes the significance of how an individual controls one’s resources and can relate to the conservation of resources theory [20], which explains that a loss of resources can affect an individual’s performance because missed information and the lack of working relationships with organizational members and functional support negatively influence goal achievement. This pragmatic approach can set apart the effects of workplace ostracism with other negative workplace behaviors such as harassment, bullying, and incivility because ostracism can have a more significant practical influence which emphasizes the loss of resources [4]. Moreover, the study found task interdependence to significantly moderate the relationship between TMX and task performance and to moderate the mediated relationship. Since high task interdependence involves frequent interactions and exchanges between individuals, ostracized individuals are likely to have reduced or limited access to organizational resources such as essential information and social relations which then negatively impacts task performance. Therefore, the study findings contribute to the literature as the moderated mediation model found workplace ostracism to go beyond the psychological effects that link workplace ostracism with behavioral outcomes and by suggesting that the indirect effects of workplace ostracism via TMX differ at the level of task interdependence.

Studies have found workplace ostracism to result in various negative attitudinal and behavioral outcomes that can greatly affect organizational performance. It is critical that organizations and managers address this issue as the study results suggest that ostracized individuals negatively perceive their social exchanges with their team members which then decreases performance. Since teams are frequently implemented in organizations, trust among team members, team cohesiveness, and team efficacy are essential for effective teamwork. In order to achieve successful performance, organizations and managers need to continuously emphasize the importance of quality interpersonal relationships and should provide a healthy working environment for creating and maintaining quality interpersonal exchanges. In this regard, organizational culture is a vital component as it can prioritize and strengthen the need of positive working relationships. For instance, setting organizational norms can be effective as organizations that address conflict and communicate more openly may be helpful in mitigating the negative effects of ostracism. Moreover, organizations should conduct team building exercises in order to build trust and increase understanding and cohesiveness among organizational members.

Although the study did not examine the source of ostracism, the study can generally suggest that workplace ostracism can affect both involved and uninvolved individuals. Unfortunately, workplace ostracism appears to affect an individual in a broader scope rather than be focused toward the source of ostracism. Although team members may or may not be the source of ostracism, all team members can be negatively affected by an ostracized individual. Thus, organizations and managers must realize the extensive effects of workplace ostracism and appropriately monitor and manage employee relationships.

### 5.2. Limitations and Future Directions

This study has a few limitations to mention. First, the study’s findings may not be generalizable as the study was conducted in South Korea. As a collectivistic society that emphasizes solidarity, concern for others, and close interpersonal relationships, workplace ostracism may be perceived differently from societies that are more individualistic and focused on individual accomplishments. Second, although the study was conducted using two-wave questionnaires, the study was cross-sectional in nature, and further, as the T2 questionnaires measured TMX, task interdependence, and task performance, it did not separate the mediating variable and the dependent variable. In addition, all of the measures were self-rated; hence, common method variance can be of concern and research can be further strengthened with longitudinal data in order to further validate the causal relationships. However, a common latent factor test was conducted according to Podsakoff et al. [27] and the results explained 34% in common variance; thus, common method bias was not a serious concern. Future studies should consider the use of multi-raters such as supervisors especially for performance behaviors such as organizational citizenship behavior and job performance. Third, studies suggested that TMX and task interdependence are group level phenomena [35]. However, the current study investigated TMX and task interdependence at the individual level of analysis and examined their effects on individual task performance rather than group level outcomes such as team performance. Although it may be sound that the variables should be examined at the group level of analysis, research has argued that TMX and task interdependence can also influence individual attitudes and behaviors [23]. In addition, there are numerous studies that have empirically investigated TMX with workplace outcomes at the individual level of analysis [18,19].

Teamwork is considered to be an integral component within organizations and to be a vital aspect for individual and organizational performance. Since interpersonal relationships significantly affect workplace attitudes and behaviors, workplace ostracism’s occurrence and impact can become an important concern that organizations must confront. As workplace ostracism research has yet to be comprehensively developed [5], future studies should continue exploring and understanding the mechanisms within workplace ostracism. First, research should investigate why ostracized individuals generally retaliate to all individuals rather than to those who are only involved. The source of ostracism can be a significant facet because the multi-foci perspective argues that different sources result in foci-specific attitudes and behaviors. For example, Wan, et al. [36] differentiated the source of workplace ostracism (supervisor and coworker) and reported that the source can differently influence customers’ service perceptions. Future studies need to investigate whether supervisor or coworker ostracism can differently impact organizational members. Second, ostracism can come in various forms (e.g., silent treatment, exile) and may result in different consequences as different forms can differ in severity such as partial ostracism (ostracized by one individual or a few individuals) or full ostracism (ostracized by larger numbers of people). Third, past research has generally focused on the outcomes of ostracism. Future research should also investigate boundary conditions that can help individuals cope with workplace ostracism. Praise and recognition from their supervisors or organization may help decrease the negative effects of workplace ostracism or allow individuals to cope with ostracism. Last, according to Robinson et al. [4], studies should examine various organizational antecedents that affect ostracism. For example, diversity should be carefully approached because although diversity is argued to create many organizational advantages, it may also be a root cause of workplace ostracism due to different values and beliefs that may increase interpersonal conflict. Williams and O’Reilly [37] found that diversity negatively affects social integration, communication, and conflict; therefore, when individuals feel they are different from others, they may perceive to be excluded or left out by others.

## 6. Conclusions

As the workplace has also become a social context where ostracism exists, this study found that the quality of exchanges among team members mediates the relationship between workplace ostracism and task performance. This study aligns with Robinson et al.’s [4] conceptual framework of how pragmatic facets associate workplace ostracism and behavioral outcomes. Furthermore, task interdependence was found to moderate the mediated relationship, thus supporting the moderated mediation model, and thereby extending the literature on workplace ostracism.

## Figures and Tables

**Table 1:** Means, standard deviations, and zero-order correlations.

	Mean	*SD*	1	2	3	4	5	6	7	8	9	10
1.Gender	0.28	0.45										
2.Age	35.17	7.66	−0.45 **									
3.Education	2.83	0.74	0.00	0.18 **								
4.Position	3.02	1.86	−0.25 **	0.67 **	0.22 **							
5.Tenure	6.42	6.62	−0.28 **	0.79 **	0.19 **	0.56 **						
6.Team tenure	4.07	4.46	−0.21 **	0.57 **	0.14 *	0.47 **	0.66 **					
7.Workplace ostracism	1.52	0.82	−0.17 **	0.30 **	0.13 *	0.15 *	0.23 **	0.12				
8.Team–member exchange	5.14	0.81	−0.11	0.04	−0.07	−0.01	−0.04	−0.03	−0.28 **			
9.Task interdependence	5.09	0.90	−0.12	0.08	−0.06	−0.07	0.03	0.00	−0.16 *	0.34 **		
10.Task performance	5.42	0.83	−0.23 **	0.33 **	0.07	0.22 **	0.25 **	0.18 **	−0.15 *	0.36 **	0.26 **	

Note. *n* = 242. * *p* < 0.05; ** *p* < 0.01.

**Table 2:** Regressions results for mediation.

Variable		β		SE	*t*	*p*	CI
		Direct and Total Effects					
Task performance regressed on workplace ostracism		−0.29		0.06	−4.59	0.000	−0.42, −0.17
TMX regressed on workplace ostracism		−0.33		0.06	−5.10	0.000	−0.46, −0.20
Task performance regressed on TMX, controlling for workplace ostracism		0.28		0.06	4.53	0.000	0.18, 0.43
Task performance regressed on workplace ostracism, controlling for TMX		−0.17		0.06	−2.64	0.009	−0.32, −0.07
R^2^ = 0.27							
	M	SE	LL 95% CI	UL 95% CI			
Bootstrap results for indirect effect							
Effect	−0.09	0.03	−0.17	−0.05			

Note. Bootstrap size = 5000. LL = lower limit: CI = confidence interval; UL = upper limit.

**Table 3:** Regression results for moderation.

	Task Performance		
	Step 1	Step 2	Step 3
Variable			
Gender	−0.10	−0.06	−0.06
Age	0.29 *	0.22	0.24 *
Education	0.02	0.04	0.04
Position	0.01	0.04	0.04
Tenure	−0.01	0.03	0.03
Team tenure	0.00	0.01	−0.01
TMX		0.31 ***	0.31 ***
Task interdependence		0.13 *	0.12
TMX x task interdependence			0.11 *
F	5.33 ***	10.00 ***	9.44 ***
R^2^	0.12	0.25	0.27

* *p* < 0.05; ** *p* < 0.01; *** *p* < 0.001.

**Table 4:** Moderated mediation results for workplace ostracism across levels of task interdependence for task performance.

		Workplace Ostracism			
		Conditional			
Moderator	Level	Indirect Effect	SE	*p*	CI
Task interdependence	Low	−0.054	0.04	0.13	−0.12, 0.01
	Mean	−0.089	0.03	0.05	−0.15, −0.04
	High	−0.126	0.03	0.00	−0.20, −0.07
